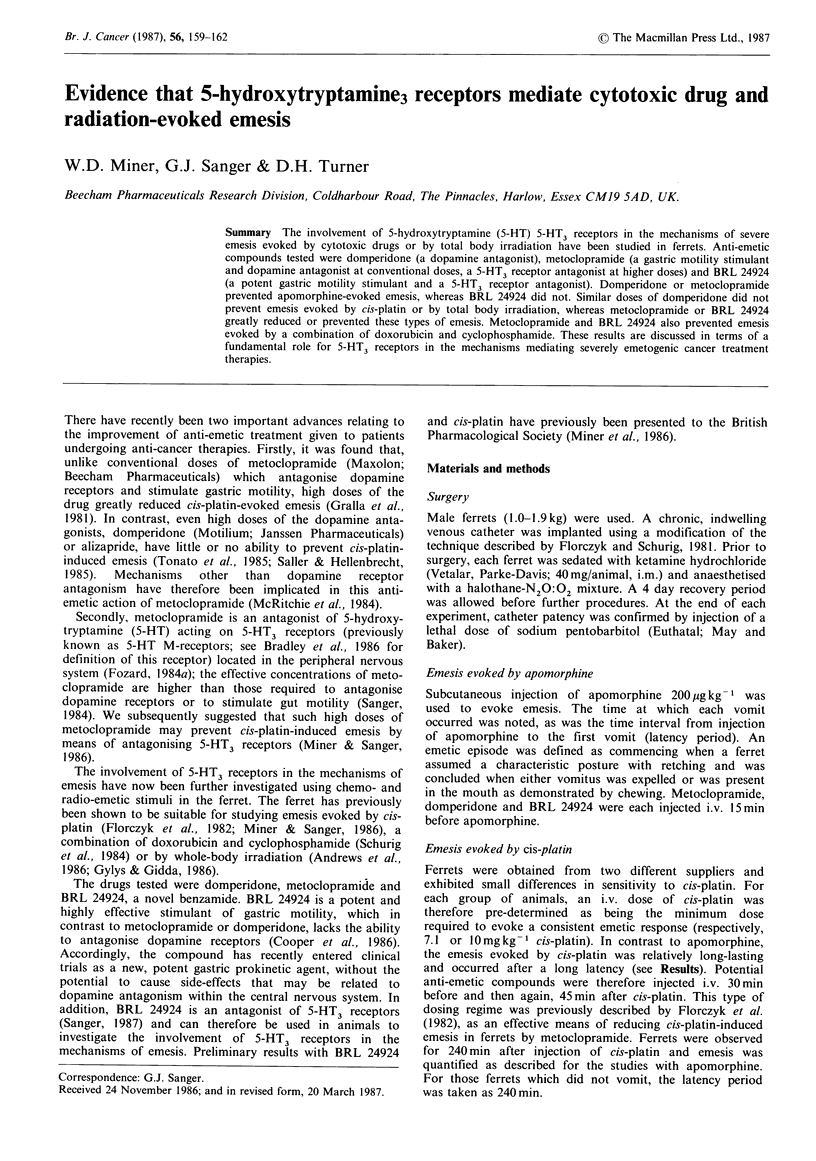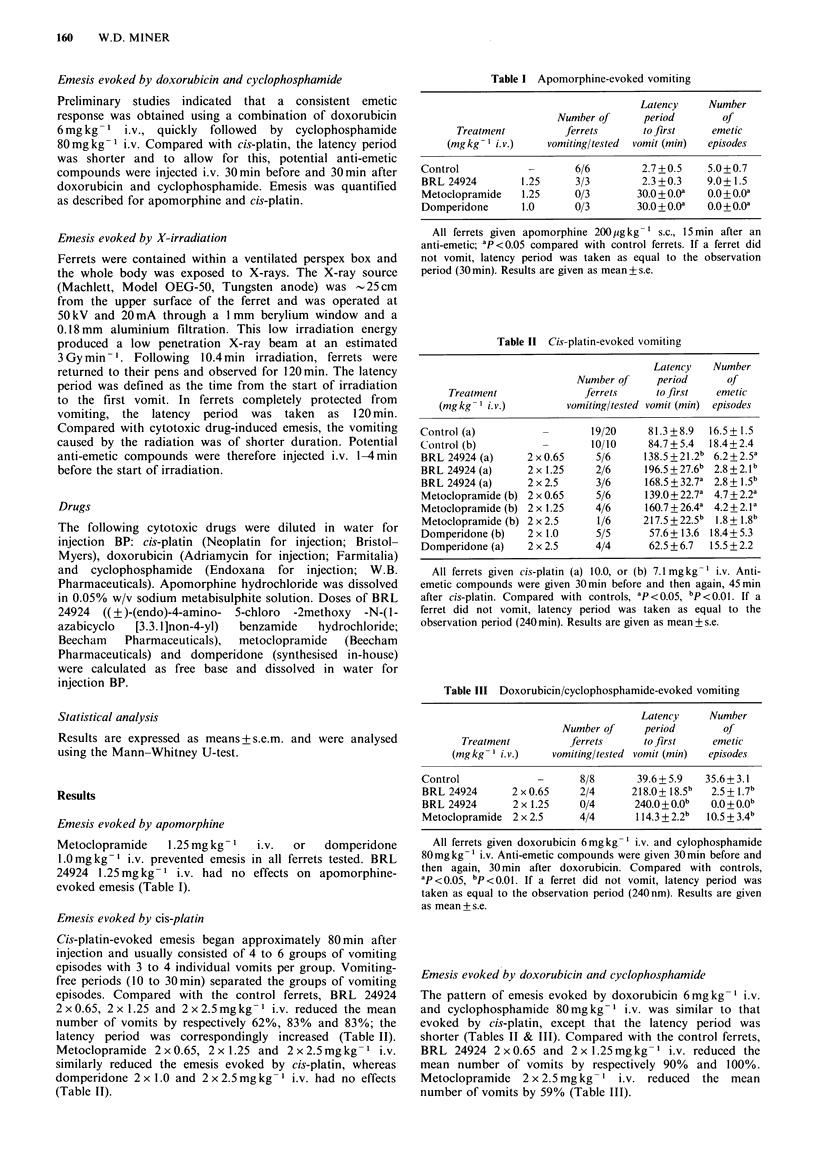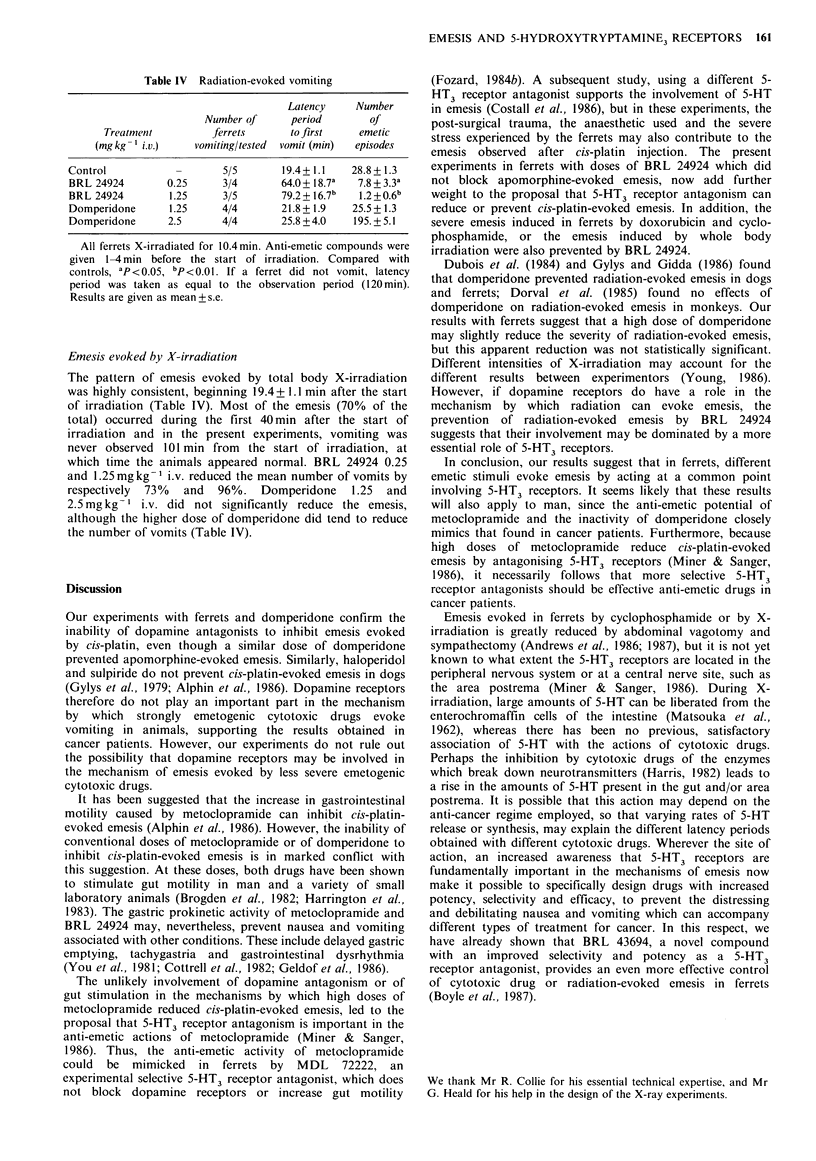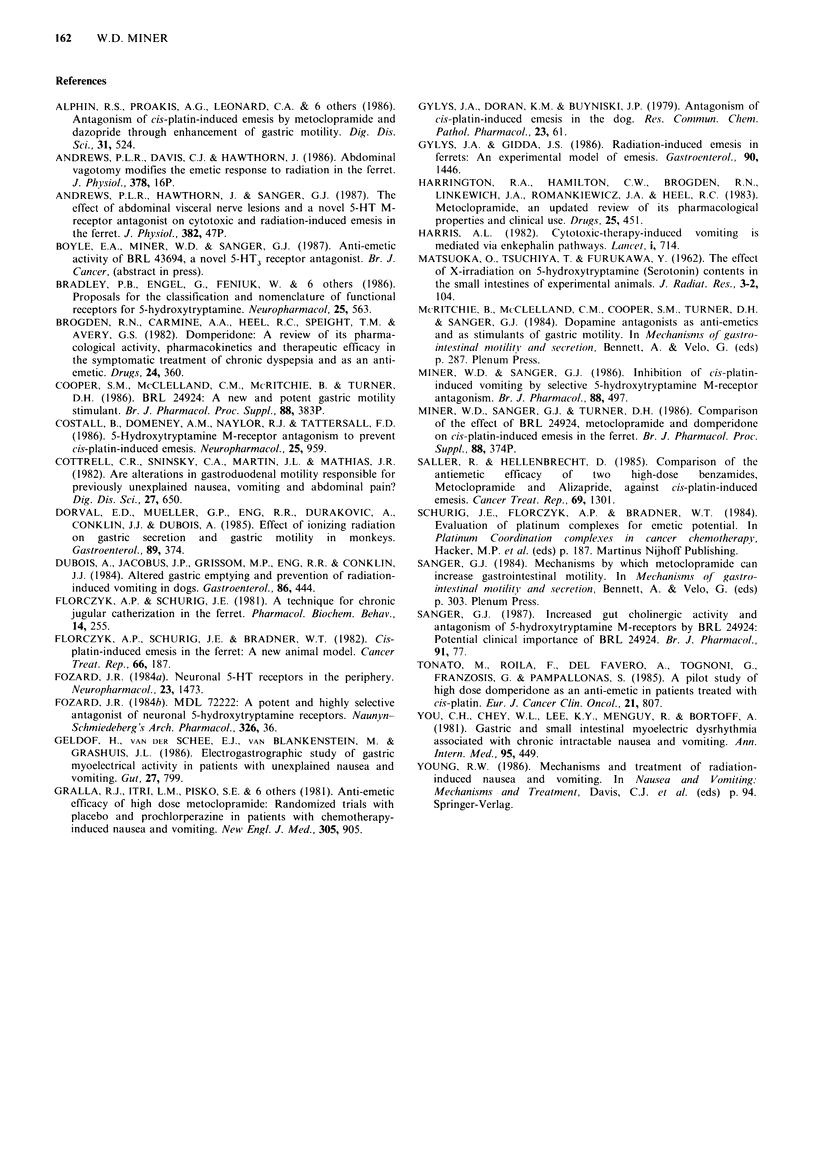# Evidence that 5-hydroxytryptamine3 receptors mediate cytotoxic drug and radiation-evoked emesis.

**DOI:** 10.1038/bjc.1987.177

**Published:** 1987-08

**Authors:** W. D. Miner, G. J. Sanger, D. H. Turner

**Affiliations:** Beecham Pharmaceuticals Research Division, Harlow, Essex, UK.

## Abstract

The involvement of 5-hydroxytryptamine (5-HT) 5-HT3 receptors in the mechanisms of severe emesis evoked by cytotoxic drugs or by total body irradiation have been studied in ferrets. Anti-emetic compounds tested were domperidone (a dopamine antagonist), metoclopramide (a gastric motility stimulant and dopamine antagonist at conventional doses, a 5-HT3 receptor antagonist at higher doses) and BRL 24924 (a potent gastric motility stimulant and a 5-HT3 receptor antagonist). Domperidone or metoclopramide prevented apomorphine-evoked emesis, whereas BRL 24924 did not. Similar doses of domperidone did not prevent emesis evoked by cis-platin or by total body irradiation, whereas metoclopramide or BRL 24924 greatly reduced or prevented these types of emesis. Metoclopramide and BRL 24924 also prevented emesis evoked by a combination of doxorubicin and cyclophosphamide. These results are discussed in terms of a fundamental role for 5-HT3 receptors in the mechanisms mediating severely emetogenic cancer treatment therapies.


Br. J. Cancer (1987), 56, 159–162 © The Macmillan Press Ltd., 1987

### Evidence that 5-hydroxytryptamine$_3$ receptors mediate cytotoxic drug and radiation-evoked emesis

W.D. Miner, G.J. Sanger & D.H. Turner

*Beecham Pharmaceuticals Research Division, Coldharbour Road, The Pinnacles, Harlow, Essex CM19 5AD, UK.*

**Summary** The involvement of 5-hydroxytryptamine (5-HT) 5-HT$_3$ receptors in the mechanisms of severe emesis evoked by cytotoxic drugs or by total body irradiation have been studied in ferrets. Anti-emetic compounds tested were domperidone (a dopamine antagonist), metoclopramide (a gastric motility stimulant and dopamine antagonist at conventional doses, a 5-HT$_3$ receptor antagonist at higher doses) and BRL 24924 (a potent gastric motility stimulant and a 5-HT$_3$ receptor antagonist). Domperidone or metoclopramide prevented apomorphine-evoked emesis, whereas BRL 24924 did not. Similar doses of domperidone did not prevent emesis evoked by *cis*-platin or by total body irradiation, whereas metoclopramide or BRL 24924 greatly reduced or prevented these types of emesis. Metoclopramide and BRL 24924 also prevented emesis evoked by a combination of doxorubicin and cyclophosphamide. These results are discussed in terms of a fundamental role for 5-HT$_3$ receptors in the mechanisms mediating severely emetogenic cancer treatment therapies.

There have recently been two important advances relating to the improvement of anti-emetic treatment given to patients undergoing anti-cancer therapies. Firstly, it was found that, unlike conventional doses of metoclopramide (Maxolon; Beecham Pharmaceuticals) which antagonise dopamine receptors and stimulate gastric motility, high doses of the drug greatly reduced *cis*-platin-evoked emesis (Gralla *et al.*, 1981). In contrast, even high doses of the dopamine antagonists, domperidone (Motilium; Janssen Pharmaceuticals) or alizapride, have little or no ability to prevent *cis*-platin-induced emesis (Tonato *et al.*, 1985; Saller & Hellenbrecht, 1985). Mechanisms other than dopamine receptor antagonism have therefore been implicated in this anti-emetic action of metoclopramide (McRitchie *et al.*, 1984).

Secondly, metoclopramide is an antagonist of 5-hydroxytryptamine (5-HT) acting on 5-HT$_3$ receptors (previously known as 5-HT M-receptors; see Bradley *et al.*, 1986 for definition of this receptor) located in the peripheral nervous system (Fozard, 1984*a*); the effective concentrations of metoclopramide are higher than those required to antagonise dopamine receptors or to stimulate gut motility (Sanger, 1984). We subsequently suggested that such high doses of metoclopramide may prevent *cis*-platin-induced emesis by means of antagonising 5-HT$_3$ receptors (Miner & Sanger, 1986).

The involvement of 5-HT$_3$ receptors in the mechanisms of emesis have now been further investigated using chemo- and radio-emetic stimuli in the ferret. The ferret has previously been shown to be suitable for studying emesis evoked by *cis*-platin (Florczyk *et al.*, 1982; Miner & Sanger, 1986), a combination of doxorubicin and cyclophosphamide (Schurig *et al.*, 1984) or by whole-body irradiation (Andrews *et al.*, 1986; Gylys & Gidda, 1986).

The drugs tested were domperidone, metoclopramide and BRL 24924, a novel benzamide. BRL 24924 is a potent and highly effective stimulant of gastric motility, which in contrast to metoclopramide or domperidone, lacks the ability to antagonise dopamine receptors (Cooper *et al.*, 1986). Accordingly, the compound has recently entered clinical trials as a new, potent gastric prokinetic agent, without the potential to cause side-effects that may be related to dopamine antagonism within the central nervous system. In addition, BRL 24924 is an antagonist of 5-HT$_3$ receptors (Sanger, 1987) and can therefore be used in animals to investigate the involvement of 5-HT$_3$ receptors in the mechanisms of emesis. Preliminary results with BRL 24924 and *cis*-platin have previously been presented to the British Pharmacological Society (Miner *et al.*, 1986).

Correspondence: G.J. Sanger.
Received 24 November 1986; and in revised form, 20 March 1987.

### Materials and methods

### *Surgery*

Male ferrets (1.0–1.9 kg) were used. A chronic, indwelling venous catheter was implanted using a modification of the technique described by Florczyk and Schurig, 1981. Prior to surgery, each ferret was sedated with ketamine hydrochloride (Vetalar, Parke-Davis; 40 mg/animal, i.m.) and anaesthetised with a halothane-$N_2O$:$O_2$ mixture. A 4 day recovery period was allowed before further procedures. At the end of each experiment, catheter patency was confirmed by injection of a lethal dose of sodium pentobarbitol (Euthatal; May and Baker).

### *Emesis evoked by apomorphine*

Subcutaneous injection of apomorphine 200 $\mu g\,kg^{-1}$ was used to evoke emesis. The time at which each vomit occurred was noted, as was the time interval from injection of apomorphine to the first vomit (latency period). An emetic episode was defined as commencing when a ferret assumed a characteristic posture with retching and was concluded when either vomitus was expelled or was present in the mouth as demonstrated by chewing. Metoclopramide, domperidone and BRL 24924 were each injected i.v. 15 min before apomorphine.

### *Emesis evoked by* cis-*platin*

Ferrets were obtained from two different suppliers and exhibited small differences in sensitivity to *cis*-platin. For each group of animals, an i.v. dose of *cis*-platin was therefore pre-determined as being the minimum dose required to evoke a consistent emetic response (respectively, 7.1 or 10 $mg\,kg^{-1}$ *cis*-platin). In contrast to apomorphine, the emesis evoked by *cis*-platin was relatively long-lasting and occurred after a long latency (see **Results**). Potential anti-emetic compounds were therefore injected i.v. 30 min before and then again, 45 min after *cis*-platin. This type of dosing regime was previously described by Florczyk *et al.* (1982), as an effective means of reducing *cis*-platin-induced emesis in ferrets by metoclopramide. Ferrets were observed for 240 min after injection of *cis*-platin and emesis was quantified as described for the studies with apomorphine. For those ferrets which did not vomit, the latency period was taken as 240 min.

*Emesis evoked by doxorubicin and cyclophosphamide*

Preliminary studies indicated that a consistent emetic response was obtained using a combination of doxorubicin 6 mg kg$^{-1}$ i.v., quickly followed by cyclophosphamide 80 mg kg$^{-1}$ i.v. Compared with *cis*-platin, the latency period was shorter and to allow for this, potential anti-emetic compounds were injected i.v. 30 min before and 30 min after doxorubicin and cyclophosphamide. Emesis was quantified as described for apomorphine and *cis*-platin.

*Emesis evoked by X-irradiation*

Ferrets were contained within a ventilated perspex box and the whole body was exposed to X-rays. The X-ray source (Machlett, Model OEG-50, Tungsten anode) was ~25 cm from the upper surface of the ferret and was operated at 50 kV and 20 mA through a 1 mm berylium window and a 0.18 mm aluminium filtration. This low irradiation energy produced a low penetration X-ray beam at an estimated 3 Gy min$^{-1}$. Following 10.4 min irradiation, ferrets were returned to their pens and observed for 120 min. The latency period was defined as the time from the start of irradiation to the first vomit. In ferrets completely protected from vomiting, the latency period was taken as 120 min. Compared with cytotoxic drug-induced emesis, the vomiting caused by the radiation was of shorter duration. Potential anti-emetic compounds were therefore injected i.v. 1–4 min before the start of irradiation.

*Drugs*

The following cytotoxic drugs were diluted in water for injection BP: *cis*-platin (Neoplatin for injection; Bristol-Myers), doxorubicin (Adriamycin for injection; Farmitalia) and cyclophosphamide (Endoxana for injection; W.B. Pharmaceuticals). Apomorphine hydrochloride was dissolved in 0.05% w/v sodium metabisulphite solution. Doses of BRL 24924 ((±)-(endo)-4-amino- 5-chloro -2methoxy -N-(1-azabicyclo [3.3.1]non-4-yl) benzamide hydrochloride; Beecham Pharmaceuticals), metoclopramide (Beecham Pharmaceuticals) and domperidone (synthesised in-house) were calculated as free base and dissolved in water for injection BP.

*Statistical analysis*

Results are expressed as means ± s.e.m. and were analysed using the Mann–Whitney U-test.

### Results

*Emesis evoked by apomorphine*

Metoclopramide 1.25 mg kg$^{-1}$ i.v. or domperidone 1.0 mg kg$^{-1}$ i.v. prevented emesis in all ferrets tested. BRL 24924 1.25 mg kg$^{-1}$ i.v. had no effects on apomorphine-evoked emesis (Table I).

*Emesis evoked by* cis-*platin*

*Cis*-platin-evoked emesis began approximately 80 min after injection and usually consisted of 4 to 6 groups of vomiting episodes with 3 to 4 individual vomits per group. Vomiting-free periods (10 to 30 min) separated the groups of vomiting episodes. Compared with the control ferrets, BRL 24924 2 × 0.65, 2 × 1.25 and 2 × 2.5 mg kg$^{-1}$ i.v. reduced the mean number of vomits by respectively 62%, 83% and 83%; the latency period was correspondingly increased (Table II). Metoclopramide 2 × 0.65, 2 × 1.25 and 2 × 2.5 mg kg$^{-1}$ i.v. similarly reduced the emesis evoked by *cis*-platin, whereas domperidone 2 × 1.0 and 2 × 2.5 mg kg$^{-1}$ i.v. had no effects (Table II).

**Table I** Apomorphine-evoked vomiting

| *Treatment (mg kg$^{-1}$ i.v.)* | | *Number of ferrets vomiting/tested* | *Latency period to first vomit (min)* | *Number of emetic episodes* |
|---|---|---|---|---|
| Control | – | 6/6 | 2.7 ± 0.5 | 5.0 ± 0.7 |
| BRL 24924 | 1.25 | 3/3 | 2.3 ± 0.3 | 9.0 ± 1.5 |
| Metoclopramide | 1.25 | 0/3 | 30.0 ± 0.0[a] | 0.0 ± 0.0[a] |
| Domperidone | 1.0 | 0/3 | 30.0 ± 0.0[a] | 0.0 ± 0.0[a] |

All ferrets given apomorphine 200 μg kg$^{-1}$ s.c., 15 min after an anti-emetic; [a]$P < 0.05$ compared with control ferrets. If a ferret did not vomit, latency period was taken as equal to the observation period (30 min). Results are given as mean ± s.e.

**Table II** *Cis*-platin-evoked vomiting

| *Treatment (mg kg$^{-1}$ i.v.)* | | *Number of ferrets vomiting/tested* | *Latency period to first vomit (min)* | *Number of emetic episodes* |
|---|---|---|---|---|
| Control (a) | – | 19/20 | 81.3 ± 8.9 | 16.5 ± 1.5 |
| Control (b) | – | 10/10 | 84.7 ± 5.4 | 18.4 ± 2.4 |
| BRL 24924 (a) | 2 × 0.65 | 5/6 | 138.5 ± 21.2[b] | 6.2 ± 2.5[a] |
| BRL 24924 (a) | 2 × 1.25 | 2/6 | 196.5 ± 27.6[b] | 2.8 ± 2.1[b] |
| BRL 24924 (a) | 2 × 2.5 | 3/6 | 168.5 ± 32.7[a] | 2.8 ± 1.5[b] |
| Metoclopramide (b) | 2 × 0.65 | 5/6 | 139.0 ± 22.7[a] | 4.7 ± 2.2[a] |
| Metoclopramide (b) | 2 × 1.25 | 4/6 | 160.7 ± 26.4[a] | 4.2 ± 2.1[a] |
| Metoclopramide (b) | 2 × 2.5 | 1/6 | 217.5 ± 22.5[b] | 1.8 ± 1.8[b] |
| Domperidone (b) | 2 × 1.0 | 5/5 | 57.6 ± 13.6 | 18.4 ± 5.3 |
| Domperidone (a) | 2 × 2.5 | 4/4 | 62.5 ± 6.7 | 15.5 ± 2.2 |

All ferrets given *cis*-platin (a) 10.0, or (b) 7.1 mg kg$^{-1}$ i.v. Anti-emetic compounds were given 30 min before and then again, 45 min after *cis*-platin. Compared with controls, [a]$P < 0.05$, [b]$P < 0.01$. If a ferret did not vomit, latency period was taken as equal to the observation period (240 min). Results are given as mean ± s.e.

**Table III** Doxorubicin/cyclophosphamide-evoked vomiting

| *Treatment (mg kg$^{-1}$ i.v.)* | | *Number of ferrets vomiting/tested* | *Latency period to first vomit (min)* | *Number of emetic episodes* |
|---|---|---|---|---|
| Control | – | 8/8 | 39.6 ± 5.9 | 35.6 ± 3.1 |
| BRL 24924 | 2 × 0.65 | 2/4 | 218.0 ± 18.5[b] | 2.5 ± 1.7[b] |
| BRL 24924 | 2 × 1.25 | 0/4 | 240.0 ± 0.0[b] | 0.0 ± 0.0[b] |
| Metoclopramide | 2 × 2.5 | 4/4 | 114.3 ± 2.2[b] | 10.5 ± 3.4[b] |

All ferrets given doxorubicin 6 mg kg$^{-1}$ i.v. and cylophosphamide 80 mg kg$^{-1}$ i.v. Anti-emetic compounds were given 30 min before and then again, 30 min after doxorubicin. Compared with controls, [a]$P < 0.05$, [b]$P < 0.01$. If a ferret did not vomit, latency period was taken as equal to the observation period (240 nm). Results are given as mean ± s.e.

*Emesis evoked by doxorubicin and cyclophosphamide*

The pattern of emesis evoked by doxorubicin 6 mg kg$^{-1}$ i.v. and cyclophosphamide 80 mg kg$^{-1}$ i.v. was similar to that evoked by *cis*-platin, except that the latency period was shorter (Tables II & III). Compared with the control ferrets, BRL 24924 2 × 0.65 and 2 × 1.25 mg kg$^{-1}$ i.v. reduced the mean number of vomits by respectively 90% and 100%. Metoclopramide 2 × 2.5 mg kg$^{-1}$ i.v. reduced the mean number of vomits by 59% (Table III).

**Table IV** Radiation-evoked vomiting

| *Treatment* (*mg kg$^{-1}$ i.v.*) | | *Number of ferrets vomiting/tested* | *Latency period to first vomit (min)* | *Number of emetic episodes* |
|---|---|---|---|---|
| Control | – | 5/5 | 19.4 ± 1.1 | 28.8 ± 1.3 |
| BRL 24924 | 0.25 | 3/4 | 64.0 ± 18.7[a] | 7.8 ± 3.3[a] |
| BRL 24924 | 1.25 | 3/5 | 79.2 ± 16.7[b] | 1.2 ± 0.6[b] |
| Domperidone | 1.25 | 4/4 | 21.8 ± 1.9 | 25.5 ± 1.3 |
| Domperidone | 2.5 | 4/4 | 25.8 ± 4.0 | 195. ± 5.1 |

All ferrets X-irradiated for 10.4 min. Anti-emetic compounds were given 1–4 min before the start of irradiation. Compared with controls, [a]$P<0.05$, [b]$P<0.01$. If a ferret did not vomit, latency period was taken as equal to the observation period (120 min). Results are given as mean ± s.e.

*Emesis evoked by X-irradiation*

The pattern of emesis evoked by total body X-irradiation was highly consistent, beginning 19.4 ± 1.1 min after the start of irradiation (Table IV). Most of the emesis (70% of the total) occurred during the first 40 min after the start of irradiation and in the present experiments, vomiting was never observed 101 min from the start of irradiation, at which time the animals appeared normal. BRL 24924 0.25 and 1.25 mg kg$^{-1}$ i.v. reduced the mean number of vomits by respectively 73% and 96%. Domperidone 1.25 and 2.5 mg kg$^{-1}$ i.v. did not significantly reduce the emesis, although the higher dose of domperidone did tend to reduce the number of vomits (Table IV).

### Discussion

Our experiments with ferrets and domperidone confirm the inability of dopamine antagonists to inhibit emesis evoked by *cis*-platin, even though a similar dose of domperidone prevented apomorphine-evoked emesis. Similarly, haloperidol and sulpiride do not prevent *cis*-platin-evoked emesis in dogs (Gylys *et al.*, 1979; Alphin *et al.*, 1986). Dopamine receptors therefore do not play an important part in the mechanism by which strongly emetogenic cytotoxic drugs evoke vomiting in animals, supporting the results obtained in cancer patients. However, our experiments do not rule out the possibility that dopamine receptors may be involved in the mechanism of emesis evoked by less severe emetogenic cytotoxic drugs.

It has been suggested that the increase in gastrointestinal motility caused by metoclopramide can inhibit *cis*-platin-evoked emesis (Alphin *et al.*, 1986). However, the inability of conventional doses of metoclopramide or of domperidone to inhibit *cis*-platin-evoked emesis is in marked conflict with this suggestion. At these doses, both drugs have been shown to stimulate gut motility in man and a variety of small laboratory animals (Brogden *et al.*, 1982; Harrington *et al.*, 1983). The gastric prokinetic activity of metoclopramide and BRL 24924 may, nevertheless, prevent nausea and vomiting associated with other conditions. These include delayed gastric emptying, tachygastria and gastrointestinal dysrhythmia (You *et al.*, 1981; Cottrell *et al.*, 1982; Geldof *et al.*, 1986).

The unlikely involvement of dopamine antagonism or of gut stimulation in the mechanisms by which high doses of metoclopramide reduced *cis*-platin-evoked emesis, led to the proposal that 5-HT$_3$ receptor antagonism is important in the anti-emetic actions of metoclopramide (Miner & Sanger, 1986). Thus, the anti-emetic activity of metoclopramide could be mimicked in ferrets by MDL 72222, an experimental selective 5-HT$_3$ receptor antagonist, which does not block dopamine receptors or increase gut motility (Fozard, 1984*b*). A subsequent study, using a different 5-HT$_3$ receptor antagonist supports the involvement of 5-HT in emesis (Costall *et al.*, 1986), but in these experiments, the post-surgical trauma, the anaesthetic used and the severe stress experienced by the ferrets may also contribute to the emesis observed after *cis*-platin injection. The present experiments in ferrets with doses of BRL 24924 which did not block apomorphine-evoked emesis, now add further weight to the proposal that 5-HT$_3$ receptor antagonism can reduce or prevent *cis*-platin-evoked emesis. In addition, the severe emesis induced in ferrets by doxorubicin and cyclophosphamide, or the emesis induced by whole body irradiation were also prevented by BRL 24924.

Dubois *et al.* (1984) and Gylys and Gidda (1986) found that domperidone prevented radiation-evoked emesis in dogs and ferrets; Dorval *et al.* (1985) found no effects of domperidone on radiation-evoked emesis in monkeys. Our results with ferrets suggest that a high dose of domperidone may slightly reduce the severity of radiation-evoked emesis, but this apparent reduction was not statistically significant. Different intensities of X-irradiation may account for the different results between experimentors (Young, 1986). However, if dopamine receptors do have a role in the mechanism by which radiation can evoke emesis, the prevention of radiation-evoked emesis by BRL 24924 suggests that their involvement may be dominated by a more essential role of 5-HT$_3$ receptors.

In conclusion, our results suggest that in ferrets, different emetic stimuli evoke emesis by acting at a common point involving 5-HT$_3$ receptors. It seems likely that these results will also apply to man, since the anti-emetic potential of metoclopramide and the inactivity of domperidone closely mimics that found in cancer patients. Furthermore, because high doses of metoclopramide reduce *cis*-platin-evoked emesis by antagonising 5-HT$_3$ receptors (Miner & Sanger, 1986), it necessarily follows that more selective 5-HT$_3$ receptor antagonists should be effective anti-emetic drugs in cancer patients.

Emesis evoked in ferrets by cyclophosphamide or by X-irradiation is greatly reduced by abdominal vagotomy and sympathectomy (Andrews *et al.*, 1986; 1987), but it is not yet known to what extent the 5-HT$_3$ receptors are located in the peripheral nervous system or at a central nerve site, such as the area postrema (Miner & Sanger, 1986). During X-irradiation, large amounts of 5-HT can be liberated from the enterochromaffin cells of the intestine (Matsouka *et al.*, 1962), whereas there has been no previous, satisfactory association of 5-HT with the actions of cytotoxic drugs. Perhaps the inhibition by cytotoxic drugs of the enzymes which break down neurotransmitters (Harris, 1982) leads to a rise in the amounts of 5-HT present in the gut and/or area postrema. It is possible that this action may depend on the anti-cancer regime employed, so that varying rates of 5-HT release or synthesis, may explain the different latency periods obtained with different cytotoxic drugs. Wherever the site of action, an increased awareness that 5-HT$_3$ receptors are fundamentally important in the mechanisms of emesis now make it possible to specifically design drugs with increased potency, selectivity and efficacy, to prevent the distressing and debilitating nausea and vomiting which can accompany different types of treatment for cancer. In this respect, we have already shown that BRL 43694, a novel compound with an improved selectivity and potency as a 5-HT$_3$ receptor antagonist, provides an even more effective control of cytotoxic drug or radiation-evoked emesis in ferrets (Boyle *et al.*, 1987).

We thank Mr R. Collie for his essential technical expertise, and Mr G. Heald for his help in the design of the X-ray experiments.

### References

ALPHIN, R.S., PROAKIS, A.G., LEONARD, C.A. & 6 others (1986). Antagonism of *cis*-platin-induced emesis by metoclopramide and dazopride through enhancement of gastric motility. *Dig. Dis. Sci.*, **31**, 524.

ANDREWS, P.L.R., DAVIS, C.J. & HAWTHORN, J. (1986). Abdominal vagotomy modifies the emetic response to radiation in the ferret. *J. Physiol.*, **378**, 16P.

ANDREWS, P.L.R., HAWTHORN, J. & SANGER, G.J. (1987). The effect of abdominal visceral nerve lesions and a novel 5-HT M-receptor antagonist on cytotoxic and radiation-induced emesis in the ferret. *J. Physiol.*, **382**, 47P.

BOYLE, E.A., MINER, W.D. & SANGER, G.J. (1987). Anti-emetic activity of BRL 43694, a novel 5-$HT_3$ receptor antagonist. *Br. J. Cancer*, (abstract in press).

BRADLEY, P.B., ENGEL, G., FENIUK, W. & 6 others (1986). Proposals for the classification and nomenclature of functional receptors for 5-hydroxytryptamine. *Neuropharmacol*, **25**, 563.

BROGDEN, R.N., CARMINE, A.A., HEEL, R.C., SPEIGHT, T.M. & AVERY, G.S. (1982). Domperidone: A review of its pharmacological activity, pharmacokinetics and therapeutic efficacy in the symptomatic treatment of chronic dyspepsia and as an anti-emetic. *Drugs*, **24**, 360.

COOPER, S.M., McCLELLAND, C.M., McRITCHIE, B. & TURNER, D.H. (1986). BRL 24924: A new and potent gastric motility stimulant. *Br. J. Pharmacol. Proc. Suppl.*, **88**, 383P.

COSTALL, B., DOMENEY, A.M., NAYLOR, R.J. & TATTERSALL, F.D. (1986). 5-Hydroxytryptamine M-receptor antagonism to prevent *cis*-platin-induced emesis. *Neuropharmacol.*, **25**, 959.

COTTRELL, C.R., SNINSKY, C.A., MARTIN, J.L. & MATHIAS, J.R. (1982). Are alterations in gastroduodenal motility responsible for previously unexplained nausea, vomiting and abdominal pain? *Dig. Dis. Sci.*, **27**, 650.

DORVAL, E.D., MUELLER, G.P., ENG, R.R., DURAKOVIC, A., CONKLIN, J.J. & DUBOIS, A. (1985). Effect of ionizing radiation on gastric secretion and gastric motility in monkeys. *Gastroenterol.*, **89**, 374.

DUBOIS, A., JACOBUS, J.P., GRISSOM, M.P., ENG, R.R. & CONKLIN, J.J. (1984). Altered gastric emptying and prevention of radiation-induced vomiting in dogs. *Gastroenterol.*, **86**, 444.

FLORCZYK, A.P. & SCHURIG, J.E. (1981). A technique for chronic jugular catherization in the ferret. *Pharmacol. Biochem. Behav.*, **14**, 255.

FLORCZYK, A.P., SCHURIG, J.E. & BRADNER, W.T. (1982). *Cis*-platin-induced emesis in the ferret: A new animal model. *Cancer Treat. Rep.*, **66**, 187.

FOZARD, J.R. (1984*a*). Neuronal 5-HT receptors in the periphery. *Neuropharmacol.*, **23**, 1473.

FOZARD, J.R. (1984*b*). MDL 72222: A potent and highly selective antagonist of neuronal 5-hydroxytryptamine receptors. *Naunyn-Schmiedeberg's Arch. Pharmacol.*, **326**, 36.

GELDOF, H., VAN DER SCHEE, E.J., VAN BLANKENSTEIN, M. & GRASHUIS, J.L. (1986). Electrogastrographic study of gastric myoelectrical activity in patients with unexplained nausea and vomiting. *Gut*, **27**, 799.

GRALLA, R.J., ITRI, L.M., PISKO, S.E. & 6 others (1981). Anti-emetic efficacy of high dose metoclopramide: Randomized trials with placebo and prochlorperazine in patients with chemotherapy-induced nausea and vomiting. *New Engl. J. Med.*, **305**, 905.

GYLYS, J.A., DORAN, K.M. & BUYNISKI, J.P. (1979). Antagonism of *cis*-platin-induced emesis in the dog. *Res. Commun. Chem. Pathol. Pharmacol.*, **23**, 61.

GYLYS, J.A. & GIDDA, J.S. (1986). Radiation-induced emesis in ferrets: An experimental model of emesis. *Gastroenterol.*, **90**, 1446.

HARRINGTON, R.A., HAMILTON, C.W., BROGDEN, R.N., LINKEWICH, J.A., ROMANKIEWICZ, J.A. & HEEL, R.C. (1983). Metoclopramide, an updated review of its pharmacological properties and clinical use. *Drugs*, **25**, 451.

HARRIS, A.L. (1982). Cytotoxic-therapy-induced vomiting is mediated via enkephalin pathways. *Lancet*, **i**, 714.

MATSUOKA, O., TSUCHIYA, T. & FURUKAWA, Y. (1962). The effect of X-irradiation on 5-hydroxytryptamine (Serotonin) contents in the small intestines of experimental animals. *J. Radiat. Res.*, **3-2**, 104.

McRITCHIE, B., McCLELLAND, C.M., COOPER, S.M., TURNER, D.H. & SANGER, G.J. (1984). Dopamine antagonists as anti-emetics and as stimulants of gastric motility. In *Mechanisms of gastro-intestinal motility and secretion*, Bennett, A. & Velo, G. (eds) p. 287. Plenum Press.

MINER, W.D. & SANGER, G.J. (1986). Inhibition of *cis*-platin-induced vomiting by selective 5-hydroxytryptamine M-receptor antagonism. *Br. J. Pharmacol.*, **88**, 497.

MINER, W.D., SANGER, G.J. & TURNER, D.H. (1986). Comparison of the effect of BRL 24924, metoclopramide and domperidone on *cis*-platin-induced emesis in the ferret. *Br. J. Pharmacol. Proc. Suppl.*, **88**, 374P.

SALLER, R. & HELLENBRECHT, D. (1985). Comparison of the antiemetic efficacy of two high-dose benzamides, Metoclopramide and Alizapride, against *cis*-platin-induced emesis. *Cancer Treat. Rep.*, **69**, 1301.

SCHURIG, J.E., FLORCZYK, A.P. & BRADNER, W.T. (1984). Evaluation of platinum complexes for emetic potential. In *Platinum Coordination complexes in cancer chemotherapy*, Hacker, M.P. *et al.* (eds) p. 187. Martinus Nijhoff Publishing.

SANGER, G.J. (1984). Mechanisms by which metoclopramide can increase gastrointestinal motility. In *Mechanisms of gastro-intestinal motility and secretion*, Bennett, A. & Velo, G. (eds) p. 303. Plenum Press.

SANGER, G.J. (1987). Increased gut cholinergic activity and antagonism of 5-hydroxytryptamine M-receptors by BRL 24924: Potential clinical importance of BRL 24924. *Br. J. Pharmacol.*, **91**, 77.

TONATO, M., ROILA, F., DEL FAVERO, A., TOGNONI, G., FRANZOSIS, G. & PAMPALLONAS, S. (1985). A pilot study of high dose domperidone as an anti-emetic in patients treated with *cis*-platin. *Eur. J. Cancer Clin. Oncol.*, **21**, 807.

YOU, C.H., CHEY, W.L., LEE, K.Y., MENGUY, R. & BORTOFF, A. (1981). Gastric and small intestinal myoelectric dysrhythmia associated with chronic intractable nausea and vomiting. *Ann. Intern. Med.*, **95**, 449.

YOUNG, R.W. (1986). Mechanisms and treatment of radiation-induced nausea and vomiting. In *Nausea and Vomiting: Mechanisms and Treatment*, Davis, C.J. *et al.* (eds) p. 94. Springer-Verlag.